# M-commerce adoption among youths in Malaysia: Dataset article

**DOI:** 10.1016/j.dib.2022.108238

**Published:** 2022-05-02

**Authors:** WeiLee Lim, Rohana Sham, Alexa Min-Wei Loi, Enami Shion, Bernard Yan-Bin Wong

**Affiliations:** a Faculty of Business and Management, UCSI University, No. 1 Jalan Menara Gading, UCSI Heights, Cheras, Wilayah Persekutuan, Kuala Lumpur 56000, Malaysia; b UCSI Graduate Business School, UCSI University, No. 1 Jalan Menara Gading, UCSI Heights, Cheras, Wilayah Persekutuan, Kuala Lumpur 56000, Malaysia

**Keywords:** Mobile commerce, PLS-SEM, COVID-19, Technology acceptance model, Malaysian youths

## Abstract

The covid-19 pandemic which took the world by storm changed our behaviour towards m-commerce with enforced movement restrictions across the world. This dataset documents the factors of consideration among Malaysian youths (age 15 to 24 years old) in their intention to adopt m-commerce. Collected from October to November 2020, a total of 396 useable responses were finalized. The questionnaire consists of individual demographic variables and factors which influence the intention of youths to adopt m-commerce in Malaysia. The dataset of demographics and m-commerce related variables can be used to further explore the correlations and description of variables. The dataset is valuable for m-commerce service providers and future works of literature in understanding the behaviour of youths and hence increase the adoption rate of m-commerce among youths.

## Specification Table

**Table:** 

Subject	Business, Management and Decision Sciences
Specific subject area	Management of Technology and Innovation
Type of data	TableFigure
	Raw Data (.xls)
	Questionnaire Survey
	Descriptive Statistic
How data were acquired	Digital surveys. Use of google forms as means of collection. A copy of the survey is provided as a supplementary file.
Data format	RawProcessedDescriptiveInferential
Description of data collection	The data collection period was during the Covid-19 pandemic between October and November 2020. Using the snowball sampling technique, the questionnaires were distributed and referred to the youths through Google Form link. The targeted respondents are between the ages of 15 and 24 and who own a smartphone device. The data collection resulted in a total of 396 samples. The returned responses were screened for missing values and treated accordingly before further analysis is conducted.
Data source location	The data was collected in Kuala Lumpur, the capital city of Malaysia
Data accessibility	Raw data were deposited at the Mendeley database:
	Repository name: MendeleyData identification number: doi:10.17632/3pbdvrd4f2.1Direct URL to data: https://doi.org/10.17632/3pbdvrd4f2.1

## Value of the Data

• The data presented provides insights for stakeholders in understanding the factors which influence the intention of the Malaysian youths to adopt m-commerce.
• The data reveals the relationships between perceived usefulness, perceived ubiquity, perceived ease of use, perceived enjoyment and m-commerce adoption intention. Future research may reuse and draw inferences from the data for comparison.
• The role of perceived enjoyment as a mediator in the model offers m-commerce service providers an understanding of the importance of perceived enjoyment among youths.
• The data offers valuable perspectives for m-commerce service providers in developing product strategies with focus on features and functions which are sought after by the youths. Product development catered for youths can benefit the service providers with an increased m-commerce adoption rate in the youth market.
• The dataset can serve as a basis of comparison for future research on the differences between youths in Malaysia and other cultures in their intention to adopt m-commerce.

## 1 Data Description

The questionnaire using an online survey format was distributed to youths in Malaysia who are aged between 15 and 24 years and who possess a smartphone with m-commerce capabilities. The questionnaire covers data on individual demographic variables, perceived usefulness, perceived ubiquity, perceived ease of use, perceived enjoyment and intention to adopt m-commerce with 22 items that measure the related intention to adopt m-commerce factors. All measurement items utilised the five-point Likert scale ranging from “1” (strongly disagree) to “5” (strongly agree). The constructs and their measurement items are presented in Table 1. The data collected using the questionnaires are prepared into the raw data file appended with this main article as a supplementary document. A total of 396 usable responses were collected.

**Table 1:** Constructs and measurement items.

Constructs		Measurement items	Sources
Perceived ubiquity	PQ1	I find using m-commerce applications an efficient way to manage my time	[16,17]
	PQ2	I find using m-commerce application fits any location, whenever I go.	
	PQ3	I find using m-commerce application gives me the ability to overcome spatial limitations	
	PQ4	I find using m-commerce application makes my life easier	
	PQ5	I find using m-commerce application enables me to find information in any place	
	PQ6	I find using m-commerce application fits my schedule well.	

Perceived usefulness	PU1	Using m-commerce would improve my efficiency in my daily work	[18]
	PU2	Using m-commerce would save up my time	
	PU3	Using m-commerce would add to my effectiveness in my daily work	

Perceived ease of use	PEOU1	It is / might be easy to pick up m-commerce	[18,19]
	PEOU2	M-commerce is understandable and clear	
	PEOU3	M-commerce is / might be easy to use	
	PEOU4	It is easy for me to become skillful at using m‐commerce	

Perceived enjoyment	PE1	Using m‐commerce is fun	[19]
	PE2	Using m‐commerce is pleasant.	
	PE3	Using m‐commerce is enjoyable.	
	PE4	Using m‐commerce is exciting.	

Intention to adopt m-commerce	INT1	Assume that I have access to m-commerce systems, I intend to use them	[18]
	INT2	I intend to use m-commerce if the cost is reasonable for me	
	INT3	I believe I will use m-commerce in the future	
	INT4	I believe my interest in m-commerce will increase in the future	

**Fig. 1:**
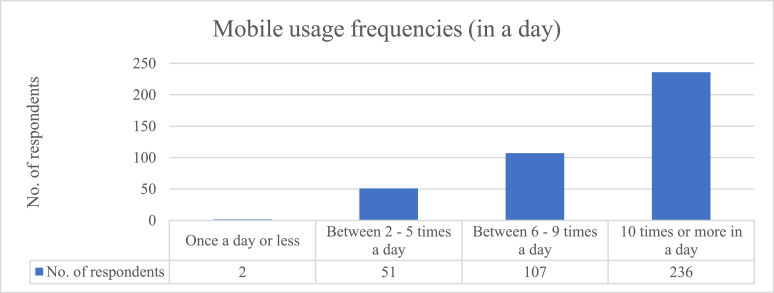
Mobile usage frequencies.

**Fig. 2:**
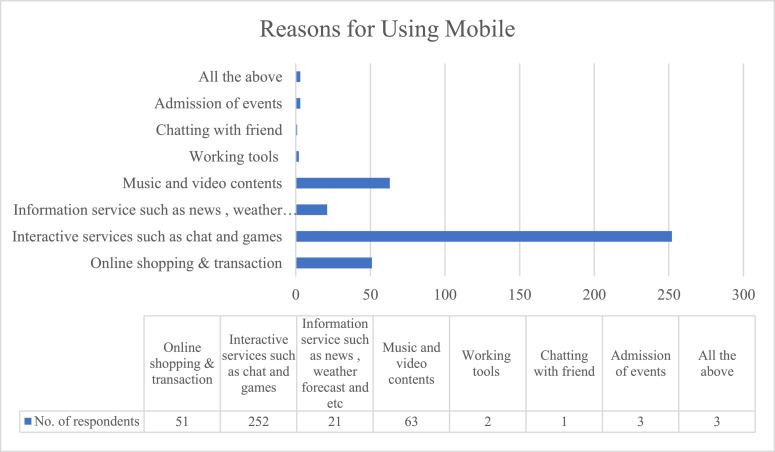
Reasons for using mobile.

**Fig. 3:**
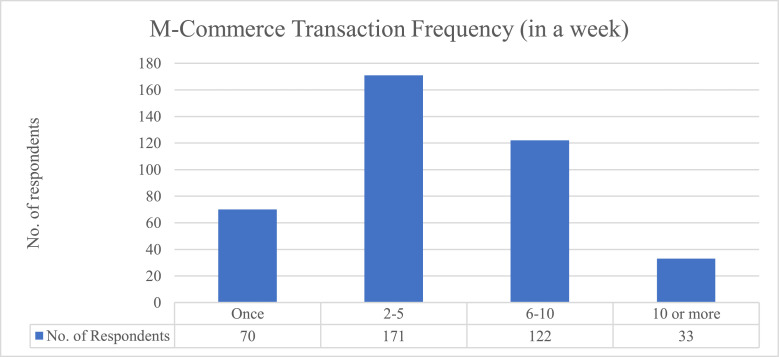
M-commerce transaction frequency (in a week).

Table 2, Fig. 1, Fig. 2, Fig. 3 illustrated the descriptive data. Table 2 presents the respondents’ demographic variables including gender, age, ethnicity, the highest level of education, mobile usage frequency in a day, reasons for using mobile, and m-commerce transaction frequency in a week. The data was analysed using frequencies and percentages.

**Table 2:** Demographics of participants (*N* = 396).

Variable	Category	Frequency	Percentage (%)
Gender	Male	209	52.78
	Female	187	47.22
Age	15–17 years old	10	2.53
	18–20 years old	135	34.09
	21–24 years old	251	63.38

Ethnicity	Malay	59	14.90
	Indian	64	16.20
	Chinese	258	65.20
	Others	15	3.80

Education	Primary or secondary certificate	83	20.96
	Diploma / advanced diploma	121	30.56
	Bachelor's degree	177	44.70
	Master's degree & above	15	3.79

Mobile usage frequency	Once a day or less	2	0.51
(In a day)	Between 2–5 times a day	51	12.88
	Between 6–9 times a day	107	27.02
	10 times or more in a day	236	59.60

Reason for use of mobile	Online shopping & transaction	51	12.88
	Interactive services such as chat and games	252	63.64
	Information services such as news, weather forecast, etc.	21	5.30
	Music and video contents	63	15.91
	Working tools	2	0.51
	Chatting with friend	1	0.25
	Admission of events	3	0.76
	All the above	3	0.76

M-Commerce transaction	Once	70	17.68
frequency (in a week)	2–5	171	43.18
	6–10	122	30.81
	10 or more	33	8.33

Results of reliability and validity analysis are shown in Table 3. Cronbach's Alpha, Dijkstra-Henseler's rho (rho_A), Compositive Reliability, and Average Variance Extracted (AVE) are indicators used to measure the reliability of all constructs [1], [2], [3]. The independent variables examined are perceived ubiquity (PQ), perceived usefulness (PU), and perceived ease of use (PEOU) while the mediator is perceived enjoyment (PE), and the dependent variable is the intention to adopt m-commerce (INT). The reliability analysis found the constructs to be above the 0.70 thresholds indicating internal consistency reliability [4], [5], [6], [7] while convergent validity is established with AVE value above 0.5 [8], [9], [10]. Meanwhile, discriminant validity is also examined using the Fornell-Larcker criterion (refer to Table 4) which requires the AVE of each construct to be compared to the squared inter-construct correlation of the same and other constructs in the model [11], [12], [13]. This is also confirmed in Table 5 displaying the individual items of the construct's outer loadings are higher than the cross-loadings of other constructs [10,14]. Issues for collinearity are also checked by examining the variance inflation factor (VIF) where constructs should display a value lower than 5 as recommended [5,15]. All constructs are well below the threshold of 5 indicating no multicollinearity issues.

**Table 3:** Reliability and validity.

					Collinearity statistics	

Constructs	Cronbach's Alpha	Dijkstra-Henseler's rho_A	Composite reliability	Average variance extracted (AVE)	Tolerance	VIF
Ease	0.811	0.818	0.876	0.640	0.445	2.245
Enjoyment	0.827	0.837	0.886	0.661	0.442	2.260
Intention	0.754	0.755	0.859	0.670		
Ubiquity	0.729	0.733	0.830	0.550	0.484	2.065
Useful	0.727	0.732	0.846	0.648	0.462	2.166

**Table 4:** Fornell-Larcker criterion.

Constructs	Ease	Enjoyment	Intention	Ubiquity	Useful
Ease	**0.800**				
Enjoyment	0.708	**0.813**			
Intention	0.634	0.681	**0.819**		
Ubiquity	0.557	0.570	0.621	**0.742**	
Useful	0.589	0.581	0.589	0.690	**0.805**

**Table 5:** Outer loadings and cross-loadings.

	Intention	Enjoyment	Ease	Ubiquity	Useful
INT1	**0.842**	0.546	0.530	0.519	0.513
INT3	**0.808**	0.520	0.494	0.484	0.441
INT4	**0.805**	0.603	0.531	0.520	0.488
PE1	0.632	**0.890**	0.627	0.491	0.544
PE2	0.465	**0.719**	0.526	0.460	0.431
PE3	0.527	**0.797**	0.589	0.464	0.444
PE4	0.579	**0.836**	0.557	0.442	0.463
PEOU1	0.587	0.600	**0.853**	0.460	0.528
PEOU2	0.402	0.566	**0.732**	0.405	0.367
PEOU3	0.519	0.527	**0.801**	0.470	0.484
PEOU4	0.508	0.572	**0.810**	0.449	0.496
PQ1	0.486	0.440	0.452	**0.713**	0.619
PQ4	0.483	0.446	0.405	**0.761**	0.477
PQ5	0.367	0.368	0.341	**0.712**	0.422
PQ6	0.487	0.427	0.441	**0.779**	0.511
PU1	0.462	0.457	0.442	0.613	**0.836**
PU2	0.494	0.387	0.413	0.527	**0.742**
PU3	0.468	0.548	0.557	0.530	**0.833**

**Fig. 4:**
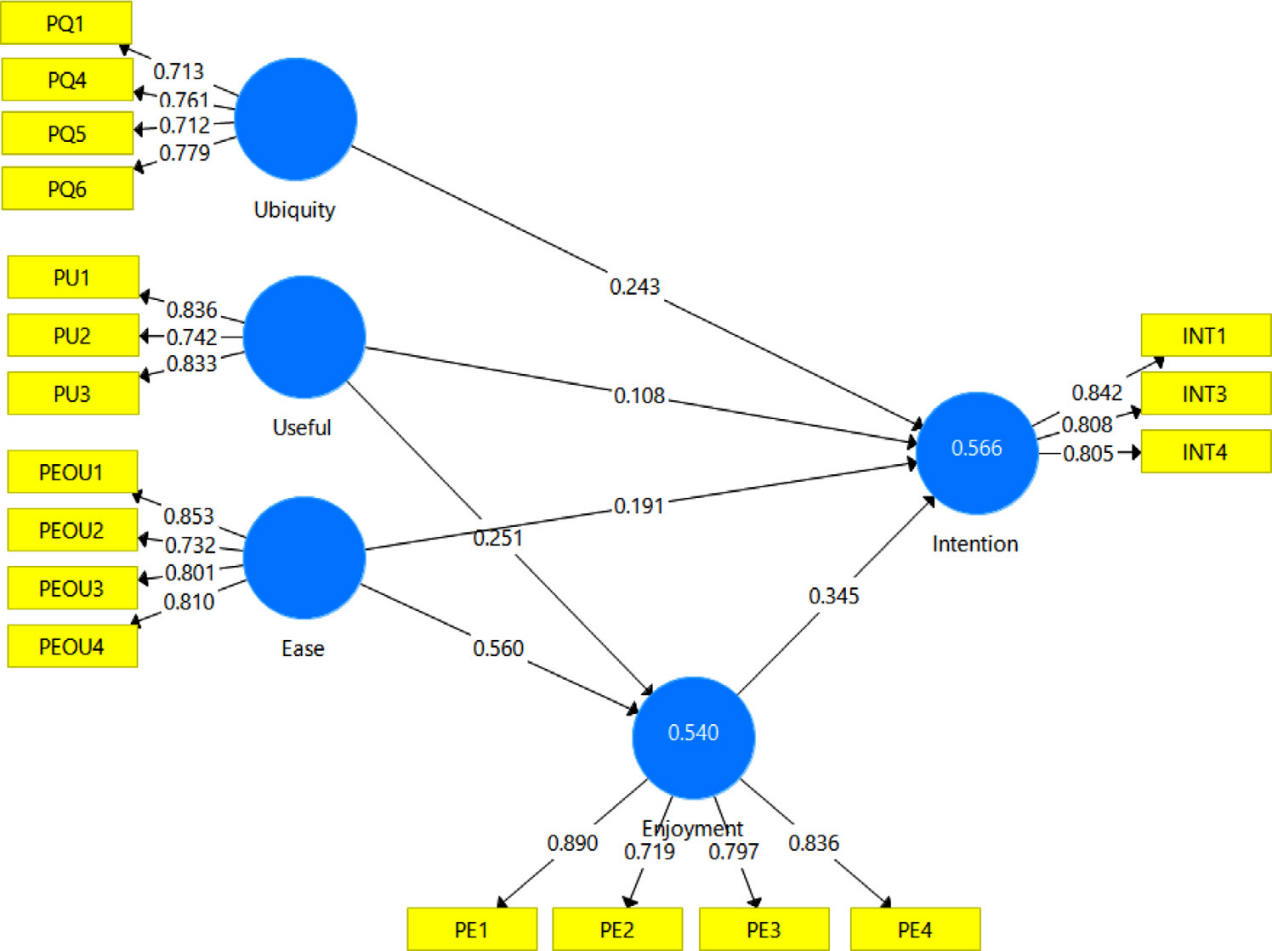
Structural model.

The analysis of the structural model is presented in Table 6 and Fig. 4. The results confirm the strength and significance of the correlations between the constructs by path analysis.

**Table 6:** Path coefficient of the variables.

Constructs	Path coefficient	T statistics	*P* Values
Ease → Enjoyment	0.560	12.510	0.000
Ease →Intention	0.191	2.799	0.005
Enjoyment → Intention	0.345	4.500	0.000
Ubiquity → Intention	0.243	4.039	0.000
Useful → Enjoyment	0.251	4.812	0.000
Useful → Intention	0.108	1.787	0.074

The mediation analysis was employed to study the relationships of perceived enjoyment as a mediator connecting perceived ease of use, perceived usefulness and intention to adopt m-commerce. The results are presented in Table 7 with total effect, direct effect and indirect effect. The bias-corrected intervals for 2.5% and 97.5% supports the mediation relationships as the spread of the interval does not contain the value of zero. Findings showed PE to fully mediate between PU and INT while showing partial mediation between PEOU and INT.

**Table 7:** Mediation analysis.

Constructs	Total effect	Direct effect	95% Bootstrapped confidence interval	Indirect effect	95% Bootstrapped confidence interval	Remarks
Ease → Enjoyment → Intention	0.384***	0.191*	(0.054, 0.324)	0.194***	(0.465, 0.644)	Partial mediation
Useful → Enjoyment → Intention	0.195**	0.108^NS^	(-0.005, 0.229)	0.087**	(0.035,0.152)	Full mediation

*Note*: ***p < 0.001, **p < 0.01, *p < 0.05.

NS *p* > 0.05.

Lastly, the structural model is evaluated for its quality with R^2^, Q^2^ and f^2^ (refer to Table 8). The R^2^value indicates the explanatory power of the structural model. As proposed by past literature [20,21], the R^2^ value of 0.75 indicates substantial relationships, 0.5 a moderate relationship and 0.25 a weak relationship [5,22,23]. The Stone–Geisser's Q^2^ indicator reflects the predictive relevance of the model and is assessed using the blindfolding procedure [14]. The threshold for Q^2^ as a rule of thumb is 0.025 as small, 0.025 to 0.50 as a medium, and above 0.50 as large [5,24]. Similarly, the f^2^ examines the significant effect of an exogenous variable on the endogenous variable. The values are evaluated using Cohen's threshold of 0.02, 0.15 and 0.35 (weak, moderate and strong) to indicate the effect size [25,26]. On predictive accuracy, the model was examined using PLSpredict (PLS prediction algorithm) to assess the PLS-SEM model and the naïve benchmark model (linear model; LM). This is performed by comparing the root mean square error (RMSE), mean absolute error (MAE) and Q^2^_predithmct_. The findings (Table 9) indicated a majority of Q^2^_predict_ to be mostly positive and both the RMSE are MAE mostly negative. The errors of the proposed model are smaller compared to a linear model. Therefore, PLS-SEM predictions establish a medium predictive power for the model.

**Table 8:** Quality of structural model.

	R square	R square adjusted	f^2^	Q^2^
Ease			0.038	
Enjoyment	0.542	0.540	0.123	0.353
Intention	0.571	0.566		0.372
Ubiquity			0.065	
Useful			0.012

**Table 9:** PLS predict assessment.

	PLS			LM			PLS-LM		
	RMSE	MAE	Q²predict	RMSE	MAE	Q²predict	RMSE	MAE	Q²predict
PE1	0.615	0.470	0.433	0.617	0.477	0.429	-0.002	-0.007	0.004
PE2	0.717	0.585	0.294	0.715	0.570	0.299	0.002	0.015	-0.005
PE3	0.700	0.544	0.355	0.704	0.543	0.347	-0.004	0.001	0.008
PE4	0.754	0.584	0.331	0.766	0.593	0.310	-0.012	-0.009	0.021
INT1	0.581	0.447	0.357	0.571	0.428	0.378	0.010	0.019	-0.021
INT3	0.627	0.464	0.295	0.628	0.465	0.294	-0.001	-0.001	0.001
INT4	0.658	0.506	0.352	0.670	0.507	0.327	-0.012	-0.001	0.025

## 2 Experimental Design, Materials and Methods

The study adopts a cross-sectional quantitative study. The unit of analysis are individuals between the age of 15 and 24 years old classified as youth and they must possess a smartphone with m-commerce functionality. Non-probability sampling technique was employed for the samples. Purposive sampling and snowball sampling was used due to the important predetermined criteria of; 1. age (between 15 and 24 years) and 2. availability of smartphone with m-commerce functionality. An online questionnaire was developed using Google Form and the link was distributed using emails, messaging apps (e.g., Whatsapp, WeChat, etc.) and social networking sites. The Google Forms were disseminated through social media with the purpose that it is better suited for youths’ behaviour. The collection period for the data was conducted from October to November 2020 during the Covid-19 pandemic. Due to the lockdown, google Forms was used as physical face-to-face was not a feasible option. As a result, a total of 396 usable responses were gathered for further analysis. The sample size of 396 exceeded the minimum sample size required as suggested by G*Power (v.3.1.9.7). The G*Power sample size calculator parameters were set at the power of 0.95, alpha value 0.05, effect size 0.15 and 4 predictors. The sample respondents are a fair representation of the youth population in Kuala Lumpur. In a study conducted by the Malaysian Communications and Multimedia Commission, the ages of 20–34 years represent the highest age group to adopt smartphones at 87%, followed by those below 20 years at 86.3% [27].

The questionnaire covers data on individual demographic variables, perceived usefulness, perceived ubiquity, perceived ease of use, perceived enjoyment and intention of adopting m-commerce among Malaysian youths. A total of 22 items adapted from previous studies measure the related intention to adopt m-commerce factors with all items utilising the Likert scale in five-point from “1” (strongly disagree) to “5” (strongly agree).

## Ethical Statement

Consent from all respondents to participate in the survey was requested and obtained through an informed consent statement included in the online survey form. In addition, anonymity was assured to the respondents with no personal data that can be identifiable to that individual. The study obtained clearance from the University's Institutional Ethics Committee and data redistribution policies of the social media platforms have been adhered to.

## CRediT authorship contribution statement

**WeiLee Lim:** Conceptualization, Methodology, Formal analysis, Writing – original draft, Writing – review & editing. **Rohana Sham:** Methodology, Formal analysis, Writing – review & editing. **Alexa Min-Wei Loi:** Methodology, Formal analysis, Writing – review & editing. **Enami Shion:** Conceptualization, Methodology, Writing – original draft, Writing – review & editing. **Bernard Yan-Bin Wong:** Conceptualization, Methodology, Writing – original draft, Writing – review & editing.

## Declaration of Competing Interest

The authors report that there is no conflict of interest to declare. There is no financial conflict of interest or any other opposing interest that may affect the study reported in this paper.

## Data Availability

M-Commerce Adoption Among Youths in Malaysia (Original data) (Mendeley Data).

## Appendix Supplementary materials

Image, application 1

Image, application 2
