# Supplementary material for: M-commerce adoption among youths in Malaysia: Dataset article
Source: Data Brief. 2022 May 2;42:108238. doi: 10.1016/j.dib.2022.108238 (PMC9111932; doi:10.1016/j.dib.2022.108238)
Supplement: Supplementary file 1 [file mmc1.docx]

**CRediT author statement**

WeiLee Lim; ***Conceptualization, Methodology, Formal Analysis, Writing - Original Draft, Writing- Reviewing and Editing***

Rohana Sham; ***Methodology, Formal Analysis, Writing- Reviewing and Editing***

Alexa Min-Wei Loi; ***Methodology, Formal Analysis, Writing- Reviewing and Editing***

Enami Shion; ***Conceptualization, Methodology, Writing - Original Draft, Writing- Reviewing and Editing***

Bernard YanBing Wong; ***Conceptualization, Methodology, Writing - Original Draft, Writing- Reviewing and Editing***
