# Supplementary material for: M-commerce adoption among youths in Malaysia: Dataset article
Source: Data Brief. 2022 May 2;42:108238. doi: 10.1016/j.dib.2022.108238 (PMC9111932; doi:10.1016/j.dib.2022.108238)
Supplement: Supplementary file 2 [file mmc2.docx]

**The Survey Dataset of
M-Commerce Adoption Among Youths in Malaysia: Dataset Article**

The authors whose names are listed immediately below certify that they have **NO** affiliations with or involvement in any organization or entity with any financial interest (such as honoraria; educational grants; participation in speakers’ bureaus; membership, employment, consultancies, stock ownership, or other equity interest; and expert testimony or patent-licensing arrangements), or non-financial interest (such as personal or professional relationships, affiliations, knowledge or beliefs) in the subject matter or materials discussed in this manuscript.

**Authors**: WeiLee Lim, Rohana Sham, Alexa Min-Wei Loi, Enami Shion and Bernard YanBing Wong
